# Dissecting molecular mechanisms underlying salt tolerance in rice: a comparative transcriptional profiling of the contrasting genotypes

**DOI:** 10.1186/s12284-019-0273-2

**Published:** 2019-03-04

**Authors:** Raheleh Mirdar Mansuri, Zahra-Sadat Shobbar, Nadali Babaeian Jelodar, Mohammad Reza Ghaffari, Ghorban-Ali Nematzadeh, Saeedeh Asari

**Affiliations:** 1 Department of Systems Biology, Agricultural Biotechnology Research Institute of Iran (ABRII), Agricultural Research, Education and Extension Organization (AREEO), PO Box 31535-1897, Karaj, Iran; 2 Department of Plant breeding and Biotechnology, Faculty of Crop Science, Sari Agricultural Science and Natural Resources University, Sari, Mazandaran 578 Iran

**Keywords:** RNA-seq, Salt stress, *Oryza sativa*, Alternative splicing

## Abstract

**Background:**

Salinity expansion in arable land is a threat to crop plants. Rice is the staple food crop across several countries worldwide; however, its salt sensitive nature severely affects its growth under excessive salinity. FL478 is a salt tolerant indica recombinant inbred line, which can be a good source of salt tolerance at the seedling stage in rice. To learn about the genetic basis of its tolerance to salinity, we compared transcriptome profiles of FL478 and its sensitive parent (IR29) using RNA-seq technique.

**Results:**

A total of 1714 and 2670 genes were found differentially expressed (DEGs) under salt stress compared to normal conditions in FL478 and IR29, respectively. Gene ontology analysis revealed the enrichment of transcripts involved in salinity response, regulation of gene expression, and transport in both genotypes. Comparative transcriptome analysis revealed that 1063 DEGs were co-expressed, while 338/252 and 572/908 DEGs were exclusively up/down-regulated in FL478 and IR29, respectively. Further, some biological processes (e.g. iron ion transport, response to abiotic stimulus, and oxidative stress) and molecular function terms (e.g. zinc ion binding and cation transmembrane transporter activity) were specifically enriched in FL478 up-regulated transcripts. Based on the metabolic pathways analysis, genes encoding transport and major intrinsic proteins transporter superfamily comprising aquaporin subfamilies and genes involved in MAPK signaling and signaling receptor kinases were specifically enriched in FL478. A total of 1135 and 1894 alternative splicing events were identified in transcripts of FL478 and IR29, respectively. Transcripts encoding two potassium transporters and two major facilitator family transporters were specifically up-regulated in FL478 under salt stress but not in the salt sensitive genotype. Remarkably, 11 DEGs were conversely regulated in the studied genotypes; for example, *OsZIFL*, *OsNAAT*, *OsGDSL,* and *OsELIP* genes were up-regulated in FL478, while they were down-regulated in IR29.

**Conclusions:**

The achieved results suggest that FL478 employs more efficient mechanisms (especially in signal transduction of salt stress, influx and transport of k^+^, ionic and osmotic homeostasis, as well as ROS inhibition) to respond to the salt stress compared to its susceptible parent.

**Electronic supplementary material:**

The online version of this article (10.1186/s12284-019-0273-2) contains supplementary material, which is available to authorized users.

## Background

Soil salinity is a major constraint for crop production in many countries. Salinity affects at least 33% of world arable lands with further areas being expected to aggravate in the coming years because of global climate changes (Zhu et al. 2015; Tester and Davenport 2003). Many countries are coping with drought where excessive evaporation leads to accumulation of salt in soil; both of these stresses can limit rice production.

Rice (*Oryza sativa*) is one of the main cereals and essential foods which provides a major source of calorie for billions of people. Rice, however, is categorized as the most saltsensitive crop plant, with an electrical conductivity (EC) threshold of 3 dSm^− 1^ for most cultivated varieties (Hoang et al. 2016), while generally, a soil is only considered saline if it has an EC > 4 dSm^− 1^ (Hoang et al. 2016). Excessive salinity challenges rice plants, particularly at the seedling and reproductive stages, often by suppressing photosynthesis and plant growth and causing biomass loss as well as partial sterility (Radanielson et al. 2018; Shahbaz et al. 2017; Hussain et al. 2018; Hossain et al. 2015). All these lead to significant reductions in major yield components. Improving salt tolerance in rice therefore is essential for ensuring food security for billions of people throughput the developing world.

Rice tolerance to salinity is genotype-dependent. A great number of salinity tolerant cultivars/landraces have been recognized with their phenotypic and physiological responses under salt stress conditions (Tahjib-Ul-Arif et al. 2018; Ali et al. 2014). Salt tolerance in rice is shown to be a polygenic trait and coordinated by multiple stress responsive genes, which also interacts with other components of stress signal transduction pathways (Reddy et al. 2017). Efforts have been made to dissect the genetics underlying salt tolerance using different methodologies. Researchers have used genome wide association studies (GWAS) to identify major genes implicated in tolerance to salinity (Chang et al. 2014; Assaha et al. 2017). Although numerous QTLs have been identified (Li et al. 2017; Quan et al. 2018), *SKC1*, encoding an HKT-type transporter, is the only gene verified to be involved in salt stress (Quan et al. 2018). Gene expression analyses using microarrays have further been used to study representation of genes implicated in the response to soil salinity in rice (Cotsaftis et al. 2011; Walia et al. 2005). Most of such studies have been based on Affymetrix genomic arrays and revealed over-representation of several salinity-induced probe sets such as genes involved in the flavonoid biosynthetic pathway. However, it is argued that these responses are related to the salt-induced damage than to enhancing tolerance. Array-based technologies, however, have critical limitations as most arrays are designed based on previously annotated genes. Nonetheless, background hybridization limits the accuracy of expression measurements, particularly for transcripts present in low abundance (Zhao et al. 2014). Following the rapid progress of massive parallel sequencing technologies, RNA sequencing has been employed to study transcriptomics in many genotypes of salt sensitive versus tolerant rice in a comparative way (Shankar et al. 2016; Razzaque et al. 2017).

The salt tolerant indica recombinant inbred line (RIL) FL478 is a novel source of salt tolerance at the seedling stage in rice (Chowdhury et al. 2016). FL478, developed from crossing between Pokkali (tolerant) and IR29 (sensitive), has shown to outperform the original Pokkali in seedling stage salinity tolerance, be photoperiod insensitive, and flower earlier than Pokkali (Cotsaftis et al. 2011). FL478 also maintains a lower Na^+^/K^+^ ratio than both parent lines. FL478 appears to be a good candidate for salinity stress tolerance in rice, particularly at the vegetative stage of growth, as it can tiller well and sustain high potassium content under salinity stress (Walia et al. 2005). Researchers have recently used RNA-seq technique to understand salt responsive mechanism in a comparison of Pokkali vs. IR29 (Shankar et al. 2016; Razzaque et al. 2017). They reported an over-representation of genes involved in MAPK signaling pathways in the tolerant genotype under salt stress. In addition, a large number of unique potassium transporters were enriched among the down-regulated genes in sensitive genotype (Shankar et al. 2016; Razzaque et al. 2017).

To get insight into the molecular mechanisms by which FL478 responds to the salt stress, we compared transcriptomic profiles of this salt tolerant genotype vs. its susceptible parent using RNA-seq technology. The investigation focused on root tissues as this organ arranges the primary defense barrier against salinity and plays a critical role in ion transport (Senadheera et al. 2009). Alternative splicing was also analyzed as it also plays a significant role in regulating gene expression in response to environmental stimuli and transcriptome diversity in plants (Kornblihtt et al. 2013). We present a panel of novel genes and transcripts differentially expressed across these two contrasting genotypes. Various metabolic pathways involved in salinity response were further revealed using functional categorization of differentially expressed transcripts. We also report several K^+^ transporters differentially spliced in the tolerant genotype compared to its susceptible parent.

## Results

### Phenotypic variability of IR29 and FL478 under salt stress

**Fig. 1:**
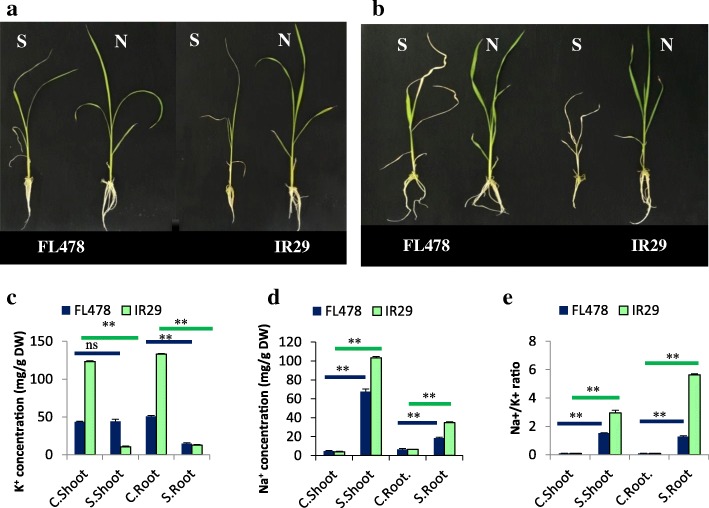
The 21-day-old seedlings of FL478 and IR29 at normal conditions and 24 h (**a**) or 1 week (**b**) after the onset of salinity stress (S:Salt, N:Normal); Concentrations of K^+^ (**c**) and Na^+^ (**d**), as well as Na^+^/K^+^ ratio (**e**) of shoots and roots in FL478 and IR29 seedlings grown under normal and salt stress conditions; Data are means ±SE. Asterisk (*) indicates significant differences between control and salt stress of the same genotype (**: *P* < 0.01, ns: not significant)

IR29 seedlings showed symptoms of salt injury after 24 h of NaCl treatment. Visual damages were observed as leaf rolling, whitish and brownish leaf tips, drying leaves, reduction in root growth and seedling height in IR29. Similar salt damages appeared in FL478, but in fewer leaves and retarded in time after salt induction. After 1 week, most FL478 seedlings remained green with continual growth, while IR29 seedlings were severely damaged in 150 mM NaCl stress (Fig. 1a, b).

At control conditions, Na^+^ concentrations of roots and shoots were similar in the salt tolerant and susceptible genotypes, while under salt stress conditions, it was distinctly higher in IR29 compared to FL478 (Fig. 1d). Further, K^+^ concentrations were maintained in shoots of FL478 under salinity stress, while it extremely decreased in IR29 (Fig. 1 c). K^+^ concentrations of roots diminished in both of the genotypes, at 150 mM NaCl, while it dropped sharper in IR29 with excessive salinity (Fig. 1 a). Consistent with a previous report (Cotsaftis et al. 2011), the Na^+^: K^+^ ratio in IR29 was higher than in FL478 under salt stress conditions (Fig. 1e).

### mRNA sequencing

To get insight into the genetics of the salt tolerance, we sought to compare genotypes of salt sensitive vs., tolerant rice at the transcriptional level. So, root RNA samples of both genotypes at 24 h after salt treatment were sequenced. Illumina paired-end sequencing generated more than 216 million raw reads from eight libraries. After filtration, 214,467,467 clean reads remained for downstream analysis. The Q20 percentage was about 97%. (Table 1, Additional file 1: Table S1). Among the total reads of FL478, 167,721,082 (76.10%) were aligned to the reference genome (including 97.40% uniquely matched and 2.59% mapped to multiple positions), while 52,671,838 (23.89%) had no match on the genome. A sum of 212,265,656 (70.97%) reads were mapped to the reference genome in IR29 (including 97.57% uniquely matched and 2.42% mapped to multiple positions), in contrast to 61,615,163 (29.02%) reads that remained unmapped (Table 1, Additional file 1: Table S1). Mapped reads were assembled in 108,633 and 109,669 transcripts, respectively in FL478 and IR29 rice cultivars (Additional file 1: Table S2). The N50 of unigenes length was 2159 and 2164 in FL478 and IR29, respectively (Additional file 1: Table S2). Further, Principle Component Analysis (PCA) was performed which revealed that two replicates were most comparable (Additional file 1: Figure S1), thus quality of throughput and sequencing was high enough for downstream analysis.

**Table 1:** Summary statistics of sequence mapping

	FL478				IR29			
Reads mapping	Control		Salinity		Control		Salinity	
	R1	R2	R1	R2	R1	R2	R1	R2
Total reads	55,577,578	60,859,580	53,881,704	50,074,058	54,337,622	54,398,510	50,651,518	52,878,006
Raw Reads	27,788,789	30,429,790	26,940,852	25,037,029	27,168,811	27,199,255	25,325,759	26,439,003
Clean Reads	27,574,497	30,227,494	26,690,719	24,842,138	26,879,937	26,904,842	25,131,953	26,215,887
Total Mapped Reads	41,551,917	46,147,036	41,297,435	38,724,694	39,700,945	39,576,373	37,632,762	33,740,413
Unique Position mapped	40,424,650	44,926,146	40,162,013	37,852,351	38,761,789	38,656,769	36,680,503	32,905,635

### Discovery of the novel transcripts through mRNA sequencing

A total of 1271 and 1430 novel transcript isoforms as well as 936 and 1031 novel transcripts were identified in FL478 and IR29 genotypes respectively (Additional file 2: Table S3). The average length of novel transcripts was 1120 bp (±863 bp)(FL478) and 1109 bp (±893 bp)(IR29). The assembled transcripts were first examined against the NR database to reveal the putative functions. Accordingly, > 58.6% and > 57.8% of the transcripts in FL478 and IR29 respectively were assigned with a putative function (Additional file 1: Figure S2). Novel transcripts corresponded to 2.03% and 2.27% of the total transcripts in FL478 in IR29 respectively (Additional file 1: Figure S2).

### Functional annotation and classification of novel transcripts

GO analysis of the novel transcripts suggested that a substantial fraction of these genes were involved in regulatory processes of transcription, gene expression, RNA metabolism, DNA repair, transport, cellular metabolic and protein modification in both genotypes. Notably, the terms of the two biological processes including ‘response to stimulus’ and ‘response to stress’ constituted the most highly represented novel transcripts in FL478 compared to IR29 (Additional file 1: Figure S3). The terms, ATP binding, DNA binding, zinc ion binding, Kinase activity, and ion binding comprised several novel transcripts in both genotypes. More than 75% of the novel transcripts were represented in nucleus, mitochondria, and plastid in both genotypes (Additional file 1: Figure S3).

### Expression profiling analyses in the tolerant and sensitive genotypes in response to salt stress

**Fig. 2:**
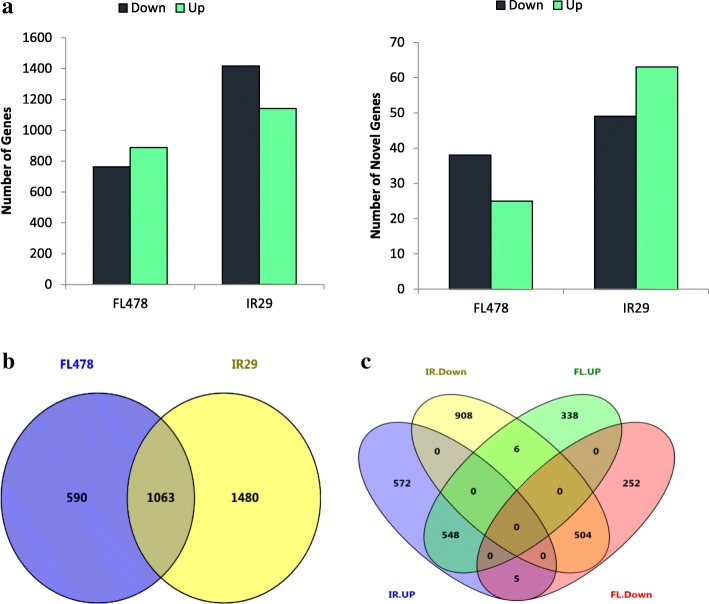
Number of genes differentially expressed under salt stress (24 h after the onset of salinity stress) in roots of the rice genotypes; **a**) The total number of genes or novels which are up- or down-regulated by salt stress; **b**) A Venn diagram of differentially expressed genes under salt stress; **c**) Number of genes expressed in common or specifically in either of the genotypes. Up: Up-regulated, Down: Down-regulated, IR: IR29, FL: FL478

To investigate transcriptional variations between tolerant and sensitive genotypes, the differentially expressed genes (DEGs) were identified under control and salt stress conditions. Consistent with the previous reports (Razzaque et al. 2017), the number of significant DEGs was higher in the salt-sensitive genotype. A total of 1714 DEGs were detected in FL478 roots, among which, 927 were up- and 787 down-regulated under salt conditions (Fig. 2a). We further found 63 novel transcripts, of which 38 were up- and 25 were down-regulated in FL478. In contrast, a total of 2670 DEGs were found in IR29 including 1204 up- and 1466 down-regulated under salt stress. Among the novel transcripts found in IR29, 63 and 49 were up- and down-regulated, respectively (Fig. 2a).

Comparative transcriptome analysis of FL478 and IR29 in response to salt stress revealed that 33.9% (*n* = 1063) of the DEGs were co-expressed in both of the genotypes (Fig. 2b). Among co-expressed DEGs, 548 and 504 DEGs were detected to be commonly up- and down-regulated, respectively in the roots of both genotypes. Further, 18.8% and 47.2% of total DEGs were specifically expressed in FL478 and IR29, respectively (Fig. 2b). A sum of 338 and 572 DEGs were exclusively up-regulated, while 252 and 908 DEGs were exclusively down-regulated in FL478 and IR29 respectively. Interestingly, 5 DEGs were down-regulated in FL478, while they were up-regulated in IR29. In contrast, 6 DEGs up-regulated in FL478 were down-regulated in IR29 (Fig. 2c). This panel of 11 DEGs therefore is suggested as promising candidate genes involved in response to salt stress (Fig. 2c). These results suggest that the tolerant genotype and its susceptible parent employ different mechanisms to respond to the salt stress.

### Gene ontology enrichment analysis of DEGs

We used the Singular Enrichment Analysis (SEA) to test the gene ontology enrichment based on the list of 1714 and 2670 DEGs in FL478 and IR29, respectively. Metabolic processes, response to stress, regulation of gene expression, as well as transport and regulation of biological processes (BP) were found as dominant terms in both genotypes. Further, some BP terms including response to abiotic stimulus, iron ion transport, cell wall macromolecule catabolic process, response to oxidative stress, response to water, and macromolecule localization were specifically enriched in FL478 up-regulated transcripts. Notably, signal transduction was significantly enriched in up-regulated transcripts of FL478, while this term was enriched in down-regulated transcript of IR29.

The most significant overrepresented molecular function (MF) terms were binding, ATP binding, kinase activity, cation transmembrane transporter activity, and transferase activity in both genotypes. However, cation binding, ion binding, zinc ion binding, and inorganic cation transmembrane transporter activity were specifically enriched in up-regulated transcripts of the tolerant genotype. In terms of cellular component (CC) ontology, the most significant enriched term was nucleus in both genotypes (Additional file 1: Figure S4a).

The enriched biological processes for down-regulated transcripts were mainly RNA processing, oxoacid metabolic process, DNA metabolic process, DNA replication, cellular process, tRNA metabolic process, and cellular carbohydrate biosynthetic process in FL478 and IR29. Furthermore, the most enriched molecular function terms of down-regulated DEGs were nucleotidyl transferase activity, catalytic activity, ATPase activity, electron carrier activity, and structural molecule activity in both genotypes, but GTP binding was found to be specific to IR29 (Additional file 1: Figure S4b).

### Identification of the significant differential transcription factors (TFs)

A total of 220 and 239 TFs encoding transcripts were found in the FL478 and IR29, respectively. Among them, 38 and 44 transcripts were differentially expressed in FL478 and IR29, respectively (Additional file 1: Figure S5). Our results revealed that most of the TF families belonged to the *MYB*, *WRKY*, *NAC*, *AP2*, *bZIP*, *HD-ZIP*, and *HSF* gene families (Additional file 1: Figure S5). Notably, *WRKY*, *AP2*, *bZIP*, *HD-ZIP*, and BHLH families were mostly enriched in FL478, while *MYB*, *NAC*, and *C2H2* families were more represented TFs in IR29 (Additional file 1: Figure S5). Interestingly, *HOX24* and *ZFP16* were the most common up-regulated families in FL478 TFs; in contrast, these TFs were highly down-regulated in IR29. Among the differentially expressed TFs, *MIKC_MADS* (*P* ≤ 1.13E-43) and *G2-likefamily* (*P* ≤ 1.64E-101) were specifically found in the tolerant genotype (FL478), which were not previously reported (Additional file 1: Figure S5).

### Metabolic pathways under salinity stress

As a complementary analysis, differentially regulated transcripts in both genotypes were further mapped to the metabolic pathways using Mapman. The overview of analyzed pathways indicated that the cell wall biosynthesis was enriched in both genotypes. In this pathway, genes encoding transport and Major Intrinsic Proteins (MIP) transporter superfamily comprising aquaporin subfamilies were specifically enriched (*P* < 0.006) in FL478. Similarly, the overview of secondary metabolite pathway indicated that the genes encoding signaling misc. and misc. cytochrome P450 (*P* < 0.02) were significantly enriched in FL478 (Additional file 1: Figure S6).

The overview of stress response pathways revealed that genes encoding for abiotic stress redox signaling, peroxidases and gluthatione S-transferases were enriched in both genotypes. Although the genes involved in signaling pathways were mapped in both genotypes, they were enriched (*P* < 0.002) only in FL478. However, the genes involved in MAPK signaling and signaling receptor kinases pathways were specifically enriched (*P* < 1.74E-7) in FL478 (Additional file 1: Figure S7).

In addition, we found that genes involved in hormone signaling pathways, including components of ABA, ethylene, and jasmonic acid were enriched in both genotypes. Also, the regulation overview revealed that genes involved intranscription regulatory containing components of *Zinc finger family*, *ABI3/VP1-related B3-domain*, and *bZIP-related transcription factor family* were enriched in FL478 and *AP2* while *HSFtranscription factor family* were enriched in IR29 (Additional file 1: Figure S8). These results suggest a notable difference in the expression of signaling pathways between IR29 and FL478.

### Identification of alternative splicing events in rice genotypes

**Fig. 3:**
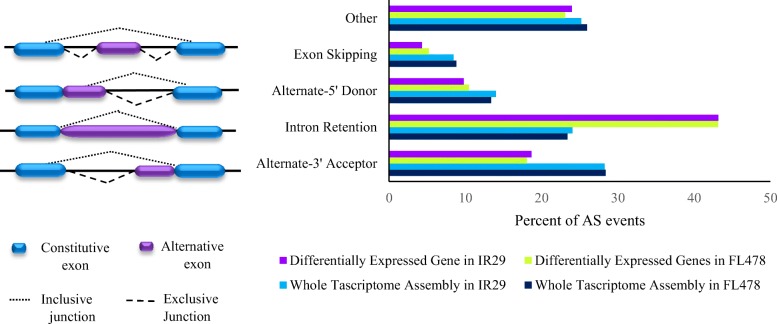
Distribution of various alternative splicing events in the transcriptomes of FL478 and IR29 under salt stress

Alternative splicing (AS) is an important factor in plants’ response to stresses because AS leads to drive more than one RNA product from a single gene and to increase transcriptional diversity (Foissac and Sammeth 2015; Shankar et al. 2016). Analysis of the AS of the total transcriptome assembly revealed that most AS events were related to the Alternative Acceptor site (AA). The corresponding figures of AS events in AA were 2592 (28.43%) and 2690 (28.27%) in FL478 and IR29 respectively (Fig. 3). The frequency of secondary dominant AS events related to Intron Retention (IR) was detected as 2134 (23.40%) in FL478 and 2288 (24.04%) in IR29 (Fig. 3). In addition, our results showed that 1221 and 1334 events happened in FL478 and IR29 respectively belonging to the Alternative Donor sites (AD). Exon skipping was found an infrequent event of the alternative splicing in both genotypes in our assembly (8%). Other complex AS events were pooled together (Fig. 3).

### Differential expression of alternatively spliced transcripts

A large number of the differentially expressed transcripts were generated through AS events. We identified a total of 1135 and 1894 AS events in isoform transcripts in FL478 and IR29, respectively. In contrast to the AAs in total transcriptome, IR (490 in FL478 and 818 in IR29) was the pre-dominant event in the DEGs in both genotypes, followed by AA, AD, and ES events. In addition, we catalogued ~ 23% of AS events as complex in FL478 and IR29. Among the rare differential AS events, AD (10.48%) and ES (5.19%) were found to be significantly more frequent in FL478 than in IR29 under salt stress (Fig. 3).

Functional classification of IRs further suggests a significant over-representation of nucleic acid binding, kinase, phosphatase, and transporter terms under salt stress in both genotypes (Additional file 1: Figure S9). The expression profiles of transcript isoforms in significant AS events were explored under salt stress to evaluate the frequency and existing diversity. A large group of transcript isoforms related to the term ‘nucleic acid binding’ including *AP2*, *WRKY*, *MYB*, and *bZIP* families were found. Also, a total of 11 transcript isoforms were recognized for LOC_Os02g27000.1 (ATP-binding and Nucleotide-binding) in our transcriptome data. Furthermore, six transcripts isoforms related to LOC_Os08g38990.1.1 (*WRKY3*0) were found to be specifically expressed in FL478, among which TCONS_00107944 was more up-regulated (log2 FC = 19.62) (Additional file 1: Figure S9a). Similarly, the transcript isoforms of AS events were related to the *AP2* family, among which TCONS_00091090 was the most significantly expressed (log2 FC = 15.95). Notably, seven transcript isoforms of *bZIP* family were identified related to LOC_Os08g26880.1, among which TCONS_00102036 was over-expressed in FL478 (log2 FC = 18.65), while it was down-regulated in IR29 (log2 FC = -1.24) (Additional file 1: Figure S9a).

Our results revealed alternative splicing events in many important genes involved in response to salt stress such as MAP kinase (MAPK) genes. For example, 10 transcript isoforms of which, four were identified for LOC_Os02g53030.1 (MAPK kinase), were specifically found in FL478, whereas they were not expressed in IR29 (Additional file 1: Figure S9 b).

We further observed six transcript isoforms encoding phosphatase, which play a potential role in salt stress tolerance (Singh et al. 2015), in FL478. Among these, TCONS_00004921 (log2 FC = 1.31) and TCONS_00004922 (log2 FC = 16.27) related to LOC_Os01g62760.1 (coding *PP2C*) were significantly enriched specifically in FL478, but not in IR29 under salt conditions (Additional file 1: Figure S9d).

Analysis of Intron Retention splicing events in this study suggested significant expression of nine unique loci encoding transporters (Additional file 1: Figure S9c). Interestingly, two transcript isoforms related to LOC_Os02g49760.1 (coding potassium transporter) were specifically up-regulated in FL478 but not in salt-sensitive genotype under salt stress. In addition, we identified two transcript isoforms TCONS_00025179 and TCONS_00025180 of the LOC_Os04g51190, encoding the major facilitator family transporters, specifically in FL478 (Additional file 1: Figure S9c). Similarly, TCONS_00091213 and TCONS_00085598, encoding peptide transporter (PTR2), were specific (log2 FC = 7) to the FL478 (Additional file 1: Figure S9c).

### Gene ontology (GO) categorization of alternatively spliced transcripts

Gene ontology (GO) analysis of dominant event IR revealed that terms including metabolic process, biological regulation, gene expression, response to stress, response to stimulus, and other regulation were significantly enriched in both genotypes, among which, metal ion transport and cation transport were found enriched exclusively in FL478 (Additional file 1: Table S4). Transcripts identified in ES and AD events were more frequent in FL478. Furthermore, in both AS events, the majority of significant categories were related to regulatory terms. Interestingly, transcripts with oxidoreductase activity, known to be implicated in abiotic stress response, were specifically enriched in FL478 (Additional file 1: Figure S10a, b).

### Validation of differential gene expression using qRT-PCR

**Fig. 4:**
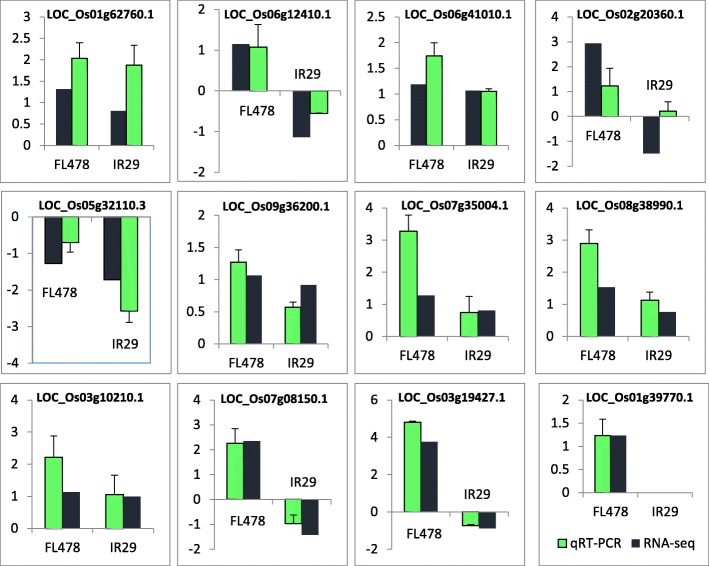
Validations of selected genes using qRT-PCR in root tissue of FL478 and IR29 under salt stress; Bar graphs depict the relative transcript abundance of the selected transcripts in the rice cultivars under different conditions. Data points are represented as log2fold change values

To further validate the RNA-Seq expression profiling, 12 salt responsive genes were nominated for qRT-PCR from different expression groups between the two contrasting genotypes including up-up, up-down, and down-down in FL478 and IR29 (Fig. 4). The qRT-PCR results were highly consistent with those of RNA sequencing in FL478 (R_2_ = 0.75) and IR29 (R_2_ = 0.85).

## Discussion

Cultivars such as FL478 are promising candidate genotypes to find the mechanisms and key genes involved in salinity tolerance which could eventually help to breed tolerant rice (Walia et al. 2005). This study aimed to decode molecular mechanisms underlying tolerance to salt stress in FL478 cultivar compared to its sensitive parent (IR29). Therefore, the whole transcriptome of FL478 and IR29 cultivars were sequenced under control and salt stress conditions.

Initially, FL478 seems to employ a molecular mechanism to punctually recognize excessive salinity. The metabolic pathways analysis revealed that genes involved in signaling pathways (containing receptor kinases and MAPK signaling) were exclusively enriched in FL478. Further, the salt induced gene coding *OsGDSL* (LOC_Os06g12410.1) in FL478 was up-regulated, while this gene was down-regulated in IR29. *OsGDSL* (*GDSL-like lipase/acylhydrolase*) is reported to be expressed in rice seedlings under different abiotic stress including salinity (Clauß et al. 2008; Lee et al. 2003, 2009; Jiang et al. 2012). In *Arabidopsis thaliana*, overexpression of *AtLTL1* coding *GDSL* was associated with elevated tolerance to salt stress (Naranjo et al. 2006). It is also reported that salicylic acid (SA) induced under salt stress leads to the overexpression of *GDSL.* The excessive *GDSL* can later release fatty acids involved in signal transduction and SA pathway, which eventually strengthens tolerance to salt stress (Jiang et al. 2012; Naranjo et al. 2006; Chaires et al. 2017)*.*

Concentrations of Na^+^, K^+^, and the Na^+^ / K^+^ ratio have been identified as the key parameters to salt stress response. The Na^+^ / K^+^ ratio in FL478 shoot was maintained by less accumulation of Na^+^ and greater K^+^ uptake, an observation consistent with the previous studies (Walia et al. 2005; Cotsaftis et al. 2011). Salt tolerant plants maintain cell ion homeostasis by ion pumps, antiporters, and carrier proteins present on the membranes (Reddy et al. 2017).

We found several transcripts encoding ion transporters responding to salt stress such as HAK25 (potassium transporter), multiple ABC transporter family, and major facilitator superfamily antiporter solely in FL478. ABC transporters are reported to affect the Na^+^/K^+^ homeostasis in Arabidopsis which improves tolerance to the salt stress (Lee et al. 2003). In this study, RCN1 (ABC transporter G family member 5) was specifically up-regulated in FL478. RCN1 is a salt tolerant factor acting via Na/k homeostasis and is up-regulated following treatment with ABA and salicylic acid (Matsuda et al. 2014).

We also identified *ZIFL*- a major facilitator superfamily antiporter - in FL478. *ZIFL*, known as a potassium transmembrane transporter, is related to auxin pathway and expresses in root tonoplast (Remy et al. 2013a; Remy et al. 2013b). In this study, we identified *HAK6* and *HAK25* specifically in FL478 with a possible involvement in tolerance to salinity. Overall, we found 18 *HAK* transporters in FL478, of which *HAK6* and *HAK25* were significantly up-regulated while *HAK4* was down-regulated. *HAK4*, the only potassium transporter encoding transcript found in IR29, was significantly down-regulated (*P* ≤ 5/00E-05). The high-affinity K^+^ uptake transporter (HAK) is a plasma membrane-bound K^+^ transporter affecting Na^+^ uptake. Some HAKs such as *HAK2* and *HAK5* were previously reported (Alemán et al. 2009; Mian et al. 2011; Wang et al. 2016; Assaha et al. 2017). A Number of genes coding aquaporin were also found to be differentially expressed in the contrasting genotypes. For example, *TIP3* and *TIP5* were found to be strongly up-regulated in FL478, while *TIP1* was the only aquaporin encoding transcript found in IR29 which was down-regulated in both genotypes. TIPs are tonoplast-expressed aquaporin generally contributing to the osmotic and turgor homeostasis in rice and Arabidopsis (Maurel et al. 1993; Maathuis et al. 2003). Notably, we observed that *OsTIP1* was down-regulated in both FL478 and IR29 genotypes, in contrast to a previous report based on microarray technology (Senadheera et al. 2009). This could be explained based on implementing different techniques or stress treatments across studies (Li et al. 2014; Lenka et al. 2011). We therefore argue that salt tolerance in FL478 can be partly due to the maintenance of ionic and osmotic homeostasis through the function of transporters and aquaporins, respectively.

We also identified transcripts encoding plant zinc finger protein (ZFP) transcription factors which were significantly up-regulated in FL478. ZFP16 and HOX24 have been reported in previous studies in abiotic stresses (Shankar et al. 2016). Based on a previous report, ZFPs reduced reactive oxygen species (ROSs) accumulation, enhanced superoxide dismutase and peroxidase activity, increased soluble sugars and proline contents, stomatal aperture, and the water loss rate, reduced K^+^ loss, decreased Na^+^ accumulation (Zang et al. 2016).

The AS events were found to be more frequent in FL478 possibly to generate various transcript isoforms in crucial regulatory processes. Abundance of AS variants in transcripts related to transporter and signaling pathway suggests their possible role in conferring stress-adaptive traits in FL478. Consistent with previous reports, the IR was the most dominant AS event in our study (Reddy et al. 2012; Wang et al. 2015; Chang et al. 2014; Shankar et al. 2016). We further observed two IR made isoform transcripts (e.g., in LOC_Os11g04020.1) related to *ZIFL*, which were up-regulated in FL478, whereas these transcripts were not expressed in IR29. *ZIFL* (Zinc-Induced Facilitator) is a tonoplast localized transporter from major facilitator superfamily, induced by drought and salinity stress (Remy et al. 2013a, b). Its probable function is proton-coupled transport of a metalchelate complex into vacuoles, and is involved in Zn and Fe homeostasis (Haydon et al. 2012).

We found that several genes of Fe uptake including *NAS1*, *NAS2*, *NAAT*, *YSL15,* and *ZIFL* were up-regulated in FL478, while they had no expression or were down-regulated in IR29. These results suggest that genes contributing to efficient Fe uptake may have been involved in salinity tolerance in FL478. The absorbable Fe declines in saline soils, so plants should have a greater capacity for absorbing Fe (Abadía et al. 2011). Three strategies are generally used by plants to absorb Fe under salt stress: acidification, reduction, and chelation. Mugineic acid (MA) was used for Fe chelation by S-adenosyl-L-methionine catalyzer with three enzymes: *nicotianamine synthase* (*NAS*), *nicotianamine aminotransferase* (*NAAT*), and *deoxymugineic acid synthase* (*DMAS*) (Itai et al. 2013; Wang et al. 2015; Yang et al. 2013). The overexpression of *NAAT* was found to confer greater amounts of MA as compared to wild-type, leading to a higher level of tolerance in transgenic rice under low Fe availability (Takahashi et al. 2001). While a partial overlap between Zn-excess and Fe-deficiency exists (Ishimaru et al. 2007), genes encoding *OsZIFL* have also been reported to implicate in the Fe-deficiency and Zn-excess pathway (Kobayashi et al. 2007; Ricachenevsky et al. 2011).

We further found that the early light-induced protein (*OsELIP*) was differentially expressed between the studied genotypes. *OsELIP* was up-regulated in FL478, while it was down- regulated in IR29. *ELIP* is a transmembrane protein, reported to be induced under high light, cold, drought, and salinity conditions (Zeng et al. 2001; Sävenstrand and Strid 2004; Tao et al. 2011). Overexpression of *ELIP* caused elevated salinity tolerance in *medicago sativa* (Zeng et al. 2001; Tzvetkova-Chevolleau et al. 2007). *ELIP* was identified as salinity-tolerance related candidate gene in seashore paspalum (*Paspalum vaginatum*), a halophytic perennial grass species (Chen et al. 2016). Under the abiotic stress, reactive oxygen species (ROS) and free electrons are generated. *ELIP* degrades ROS molecules to the compounds and free electrons which eventually turn to the common signal transduction components (Doyle et al. 2009). In rice transgenic lines, overexpression of *OsTPS1* results in greater concentrations of trehalose and proline compared to the wild type. *OsTPS1* also co-expresses with some stress-related genes including *WSI18*, *RAB16C*, *HSP70*, and *ELIP* (Li et al. 2011). We also observed that *WSI18* and *RAB16C* were specifically up-regulated in FL478. Therefore, *SGR* and *ELIP* can be among the candidate genes for salt tolerance, based on the achieved results.

## Conclusions

**Fig. 5:**
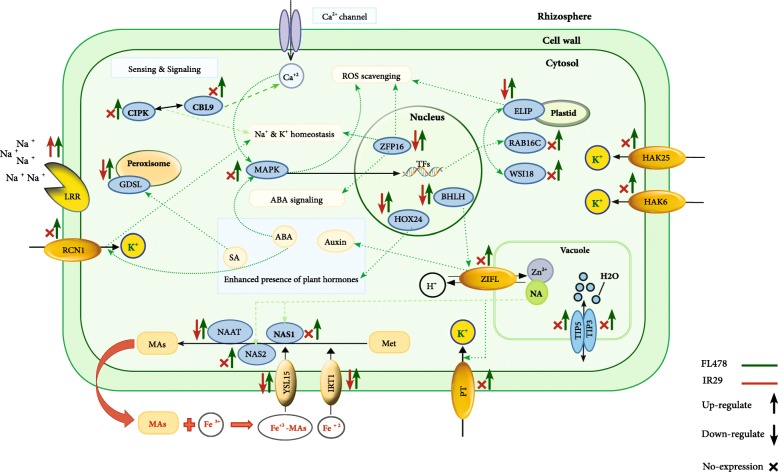
Schematic representation of the molecular response to salt stress in FL478 versus IR29; More efficient mechanisms are in place in the tolerant genotype, especially in signal transduction of salt stress, influx and transport of K^+^, ionic and osmotic homeostasis, as well as ROS inhibition to respond to the salinity as compared to its susceptible parent. (PT: Potassium Transporter; Met: Methionine; MAs: Mugineic acid)

The systematic comparison of molecular responses to salt stress in FL478, as a rice tolerant genotype, versus its susceptible parent suggested that it employs a sophisticated mechanism for responding to the salt stress (Fig. 5) including 1) Prompt signal transduction of the salt stress; possibly through enrichment of MAPKs and receptor kinases as well as some stress related TF families comprising *WRKY*, *AP2*, *bZIP*, *HD-ZIP,* and BHLH, and punctual up-regulation of some relevant genes such as *OsGDSL*, *HOX24*, and *ZFP16*; 2) Proficient influx and transport of k^+^; via up-regulation of several potassium transporters such as *HAK6*, *HAK25* and *RCN1*; 3) Maintaining ionic and osmotic homeostasis; through induction of some genes involved in Fe uptake (including *NAS1*, *NAS2*, *NAAT*, *YSL15)* and Zn homeostasis (e.g. *ZIFL* from major facilitator superfamily), as well as aquaporins e.g. *TIP3* and *TIP5*; 4) ROS inhibition; by up-regulation of *OsELIP* and some stress inducible genes such as *WSI18* and *RAB16C* with established roles in osmoprotection*.* These results provides useful information to decipher the genetics underlying the response to salinity stress, which could eventually pave the way to genetically improve this important crop for achieving salt tolerant cultivars.

## Methods

### Plant material and salinity stress treatment

The rice (*Oryza sativa* L.) seeds of cultivars IR29 and FL478 were obtained from International Rice Research Institute IRRI. The seeds were sterilized with 3–6% sodium hypochlorite solution (with one drop of Tween 20 per tube) and germinated in the dark at 28 °C in the germinator. The young seedlings were transferred to hydroponic system (plastic trays on the Styrofoam sheet containing4 L of distilled water) in the growth chamber (14 h light/10 h dark at temperature 28 ± 2 °C) for 4 days. The hydroponic experiments were performed in a factorial arrangement based on a complete randomized block design with 3 biological replicates containing 10 samples each. The seedlings were then grown in Yoshida solution (Yoshida et al. 1971) for 21 days. The treatments were applied by transferring the 21-day-old seedlings to Yoshida solution with or without 150 mM NaCl. The root samples were then collected 24 h or 1 week after the onset of salinity stress, from both control and salt-treated plants, and immediately placed in liquid nitrogen and stored at − 80 °C until RNA extraction.

### Measurements of Na^+^ and K^+^ concentrations

One week after salt stress treatment, the roots of each genotype were harvested, washed carefully by distilled water, and dried at 70 °C for 48 h. Then, Na^+^ and K^+^ concentrations were determined using flame spectrophotometry method (Lachica et al. 1973).

### RNA extraction, cDNA library synthesis, and mRNA sequencing

For RNA extraction, equal amounts of root samples from 10 individual plants of each genotype were pooled and ground. Total RNA was extracted from 100 mg of the root samples collected at 24 h after salt treatments from both control and salt-treated IR29 and FL478 plants using an RNeasy Plant kit (Qiagen). The RNA integrity and quality was checked by a NanoDrop ND-1000® spectrophotometer, agarose gel electrophoresis, and Agilent Bioanalyzer 2100 system (Agilent Technologies Co. Ltd., Beijing, China). Only samples with an RNA integrity number (RIN) > 7.6 were used for RNA sequencing. Reads of 150 bp length were generated with the IlluminaHiSeq™ 2500 sequencing platform at Novogene Bioinformatics Institute (Beijing, China) for two biological replicates of control and salt treated IR29 and FL478 root samples.

### Reads quality and RNA-seq data analysis

A preliminary raw sequencing reads in FASTQ format were quality checked by FASTQC software where high-quality reads (Phred score ≥ 20) were confirmed. The high-quality paired-end reads were then mapped against the reference genome sequences IRGSP 1.0 (https://plants.ensembl.org/info/website/ftp/index.html) using TopHat (Trapnell et al. 2012). Bowtie (Langmead et al. 2009) was used to index the genome based on the *O. sativa* cv. Nipponbare (ssp. japonica) reference genome annotation (ftp://ftp.ensemblgenomes.org/pub/release-42/plants/gtf/oryza_sativa). Then, assembly was performed viaCufflinks utility (Trapnell et al. 2012) on the TopHat-generated alignment. Finally, transcriptome assembly was done by cuffmerge meta assembler. Blastx was employed for functional annotation of the assembled transcripts with an E-value cut-off of ≤1e^− 5^ against the rice proteome sequence, downloaded from RGAP and non-redundant (NR) protein database from NCBI, using Blast2GO program (Conesa et al. 2005). For identification of novel transcripts, Cuffcompare utility in Cufflinks package was used. The annotation of novel transcripts was performed using blastx search against the NR database. Expression levels for each transcript were calculated by quantifying the reads according to the FPKM (Fragments Per Kilobase Million) method. Differentially expressed genes (DEGs) were determined using Cuffdiff, *Q-*value cut-off of ≤0.05 and log2 fold change ≥2 (up-regulated genes) and ≤ (− 2) (down-regulated genes). CummeRbund utility (http://compbio.mit.edu/cummeRbund/) R package was used for subsequent analyses (management, visualization, and integration of DEGs).

### Gene ontology enrichment

DEGs were subjected to enrichment analysis using AgriGO public web tool, after being annotated for GO terms based on molecular function, biological processes, and cellular components. The over-represented GO terms were then identified by Fisher’s exact test (*P* < 0.05) and corrected by the FDR method < 0.05 (Conesa et al. 2005).

### Biological pathway analysis of differentially expressed genes

For Pathway analysis of DEGs, MapMan (version 3.5.1; http://mapman.gabipd.org/mapman-version-3.5.1) was used with *P*-value cut-off of ≤0.05 to visualize salt stress related changes in the general metabolism (Thimm et al. 2004).

### Alternative splicing analysis

Alternative splicing was performed using AStalavista online software (version 3; http://astalavista.sammeth.net/) by setting default parameters to four basic AS events including exon skipping (ES), alternative donor site (AD), alternative acceptor site (AA), and intron retention (IR). The remaining complex AS events were then collectively grouped as another type. Each assembly file was used as input and the output of all the AS events was further analyzed manually.

### Validation of DEG by quantitative real-time RT-PCR

To validate DEGs using quantitative real-time PCR (RT-qPCR), a sum of 12 genes were randomly selected from the panel of genes responding to salinity stress identified in RNA-seq experiment. The gene specific primers (Additional file 1: Table S5) were designed using Oligo 7.0 (ver. 5.0; National Bioscience Inc.,Plymouth, USA). Synthesis of cDNA was performed using iScript™ cDNA synthesis kit (BIO-RAD) according to the manufacturer’s protocol. The qRT-PCR was performed for three independent biological replicates of root tissues from both control and salt-treated IR29 and FL478 plants, using a LightCycler® 96 Real-Time PCR System (Roche Life Science, Germany) and SYBR Premix EX TaqII (Takara Bio, Japan) based on manufacturer’s instructions. Rice ubiquitin gene (OS04G0628100) was used as an appropriate internal control gene according to the previous studies (Shankar et al. 2016; Mizuno et al. 2010). Transcript levels of the genes were computed as 2^- ΔΔCt^ (Livak and Schmittgen 2001).

## Additional files

Additional file 1: **Table S1.** Summary of sequencing results. **Table S2.** A summary of the assembly statistics. **Table S4.** Highly enriched gene ontology (GO) terms for the differentially expressed intron retention (IR). **Table S5.** List of primers used for qRT-PCR analysis. **Figure S1.** PCA analysis and the distributions of FPKM scores based on replicates. PCA of transcriptome data in FL478 (a) and IR29 (b). The cs Density plot based on the distributions of FPKM scores in FL478 (c) and IR29 (d). **Figure S2.** Grouping transcripts into annotated, un-annotated, and annotated and un-annotated novel transcripts in FL478 and IR29. **Figure S3.** GO term assignment of novel transcripts identified in IR29 and FL478 cultivars (BP: biological processes, MF: molecular function, CC: cellular component). **Figure S4.** GO classifications of (a) up-regulated and (b) down-regulated DEGs between FL478 and IR29. **Figure S5.** Transcription factor families differentially expressed in the rice cultivars under salt stresses. **Figure S6.** A visualized overview of the metabolic pathways in rice cultivars a) FL478 and b) IR29 under salt stress drown by MapMan. Color coding; red: up-regulated transcripts and blue: down-regulated transcripts.**Figure S7.** A graphical visualization of metabolic pathways involved in differentially expressed transcripts in a) FL478 and b) IR29 under salt stress drown by MapMan. Color coding; red: up-regulated transcripts and blue: down-regulated transcripts. **Figure S8.** Schematic overview of gene regulation of differentially expressed transcripts in rice cultivars under salinity stresses, as drawn by MapMan. Various pathways were enriched in FL478 rice cultivar under salinity stress (a) and IR29 rice cultivar under salinity stress (b). Color coding; red: up-regulated transcripts and blue: down-regulated transcripts. **Figure S9.** Differential expression of transcripts produced through intron retention (IR) event in FL478 and IR29. **Figure S10.** Geneontology enrichment statistics of transcripts produced through a) Exon skipping and b) Alternative donor in FL478 and IR29. (DOCX 3446 kb)

Additional file 2: **Table S3.** The list of novel transcripts in FL478 and IR29. (XLSX 214 kb)
