# Supplementary material for: Dissecting molecular mechanisms underlying salt tolerance in rice: a comparative transcriptional profiling of the contrasting genotypes
Source: Rice (N Y). 2019 Mar 4;12:13. doi: 10.1186/s12284-019-0273-2 (PMC6399358; doi:10.1186/s12284-019-0273-2)
Supplement: Supplementary file 1 — Table S1. Summary of sequencing results. Table S2. A summary of the assembly statistics. Table S4. Highly enriched gene ontology (GO) terms for the differentially expressed intron retention (IR). Table S5. List of primers used for qRT-PCR analysis. Figure S1. PCA analysis and the distributions of FPKM scores based on replicates. PCA of transcriptome data in FL478 (a) and IR29 (b). The cs Density plot based on the distributions of FPKM scores in FL478 (c) and IR29 (d). Figure S2. Grouping transcripts into annotated, un-annotated, and annotated and un-annotated novel transcripts in FL478 and IR29. Figure S3. GO term assignment of novel transcripts identified in IR29 and FL478 cultivars (BP: biological processes, MF: molecular function, CC: cellular component). Figure S4. GO classifications of (a) up-regulated and (b) down-regulated DEGs between FL478 and IR29. Figure S5. Transcription factor families differentially expressed in the rice cultivars under salt stresses. Figure S6. A visualized overview of the metabolic pathways in rice cultivars a) FL478 and b) IR29 under salt stress drown by MapMan. Color coding; red: up-regulated transcripts and blue: down-regulated transcripts.Figure S7. A graphical visualization of metabolic pathways involved in differentially expressed transcripts in a) FL478 and b) IR29 under salt stress drown by MapMan. Color coding; red: up-regulated transcripts and blue: down-regulated transcripts. Figure S8. Schematic overview of gene regulation of differentially expressed transcripts in rice cultivars under salinity stresses, as drawn by MapMan. Various pathways were enriched in FL478 rice cultivar under salinity stress (a) and IR29 rice cultivar under salinity stress (b). Color coding; red: up-regulated transcripts and blue: down-regulated transcripts. Figure S9. Differential expression of transcripts produced through intron retention (IR) event in FL478 and IR29. Figure S10. Geneontology enrichment statistics of transcripts produced through [file 12284_2019_273_MOESM1_ESM.docx]

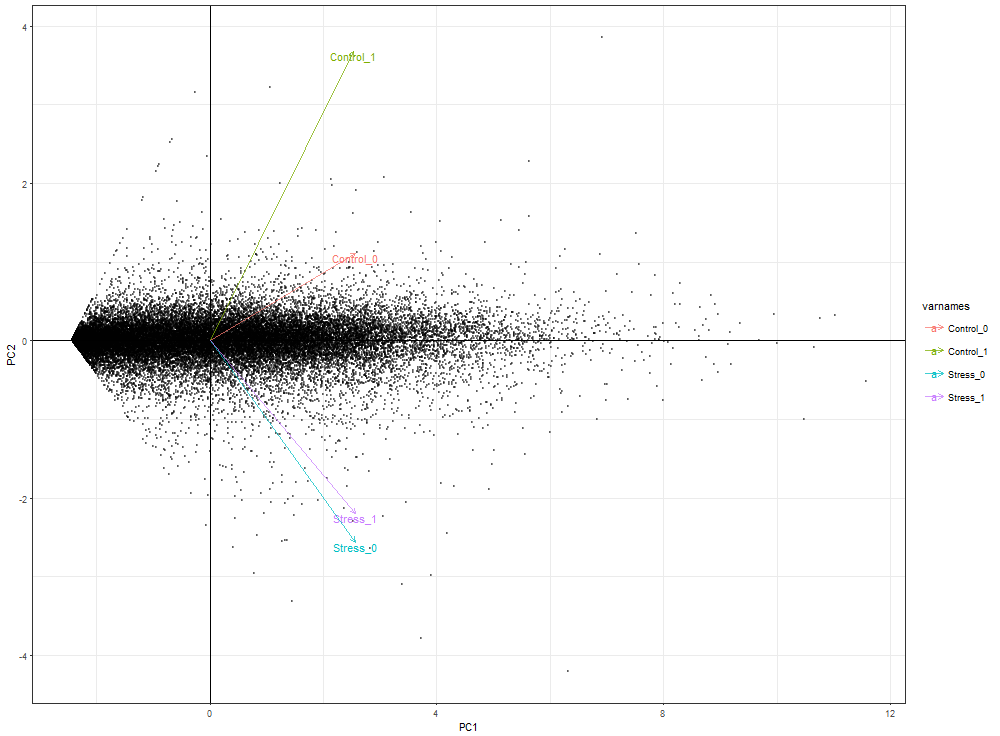


**(a)**


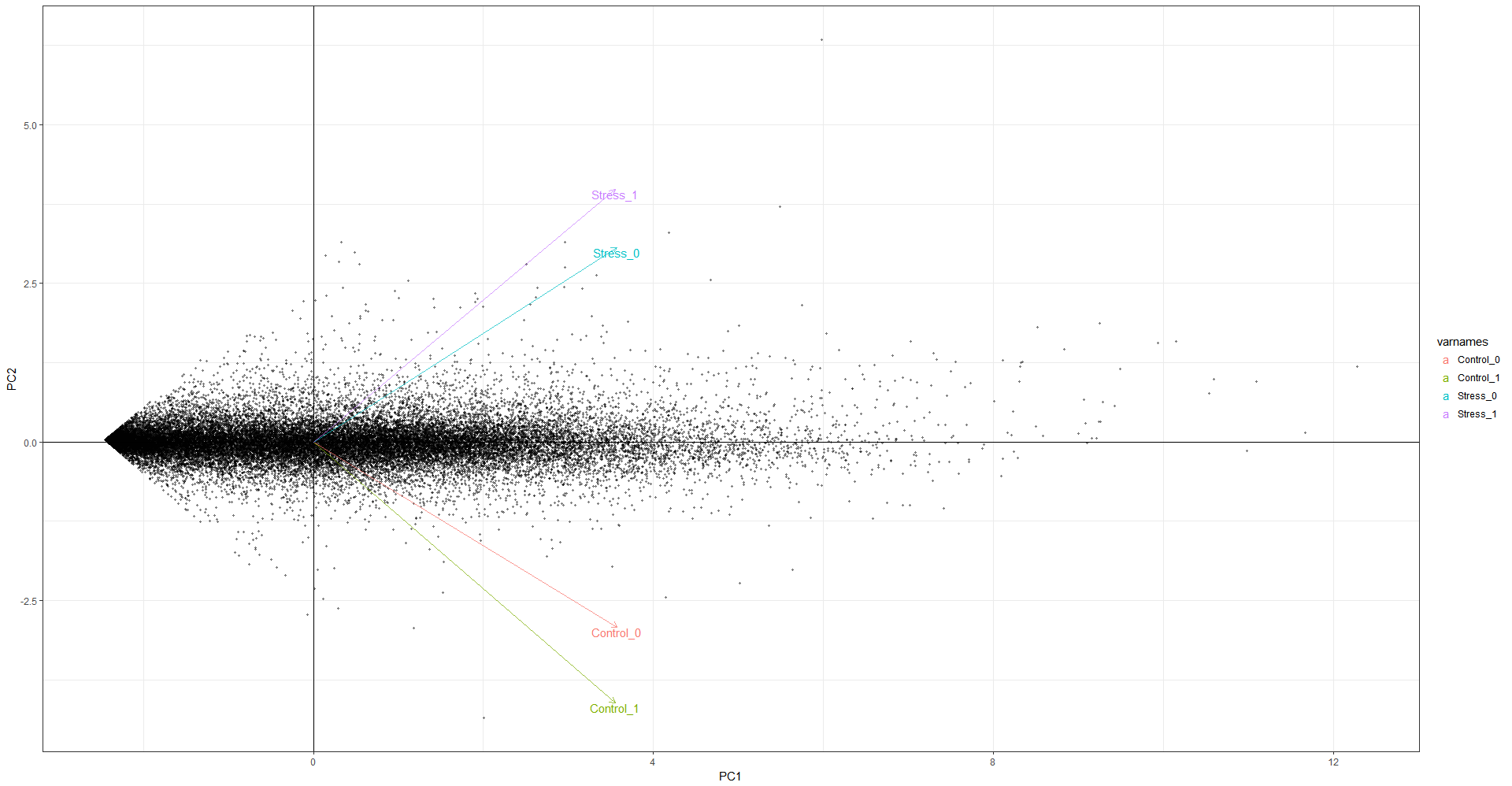


**(b)**


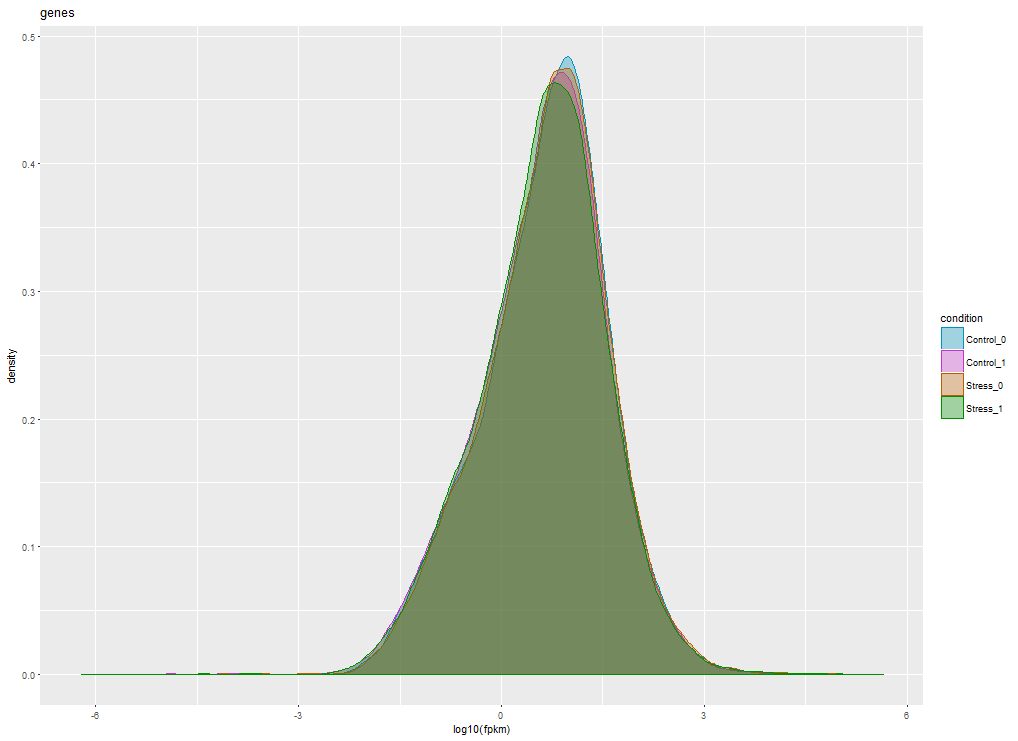

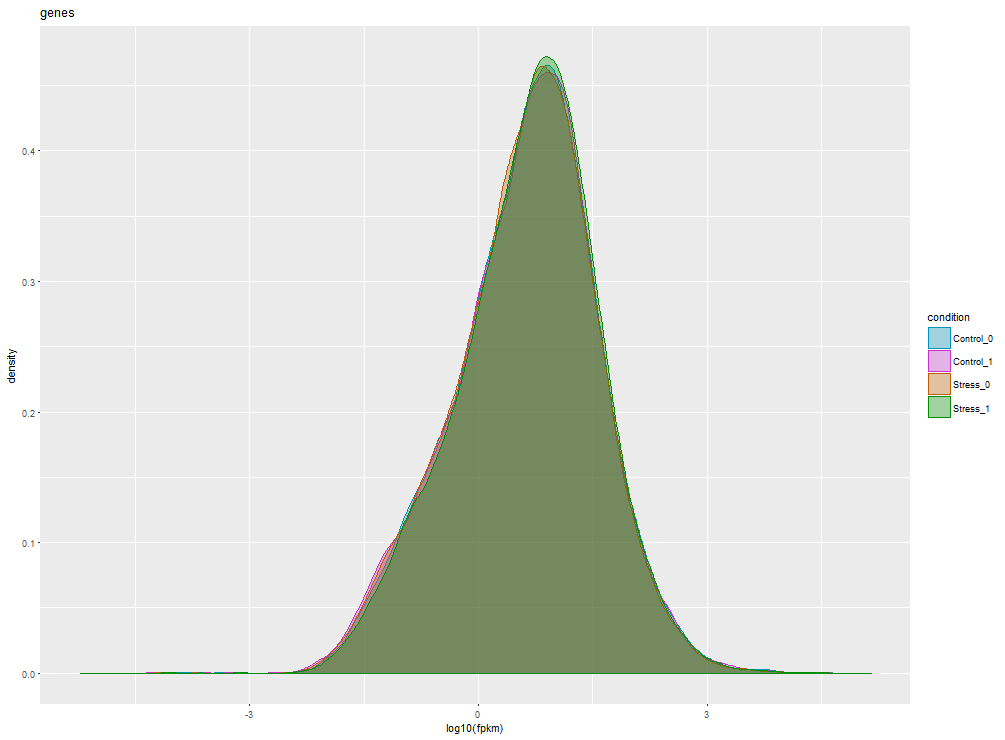


**(d)**

**(c)**

Fig S1. PCA analysis and the distributions of FPKM scores based replicates. PCA of transcriptome data in FL478 (a) and IR29 (b). The csDensity plot based on the distributions of FPKM scores­­ in FL478 (c) and IR29 (d).


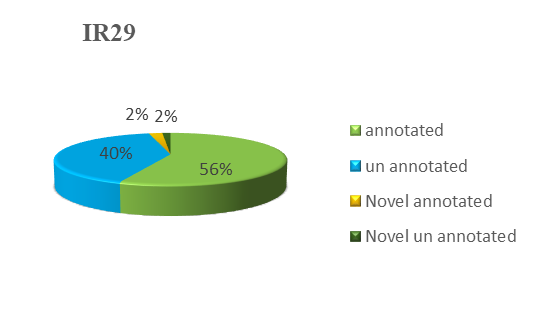

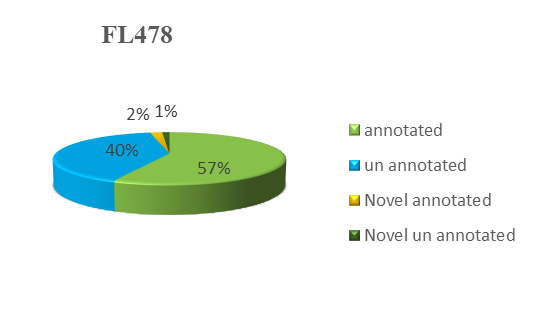


Fig S2. Grouping transcripts into annotated, un-annotated, and annotated and un-annotated novel transcripts in FL478 and IR29.

MF

CC

BP


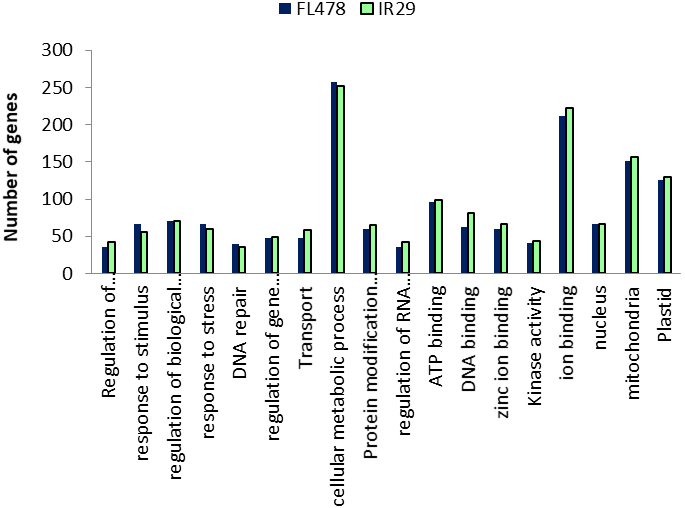


Fig S3. GO term assignment of novel transcripts identified in IR29 and FL478 cultivars (BP: biological processes, MF: molecular function, CC: cellular component).

**(a)**

**(b)**

Fig S4**.**GO classifications of (a) up-regulated and (b) down-regulated DEGs between FL478 and IR29.

Fig S5. Transcription factor families differentially expressed in the rice cultivars under salt stresses.


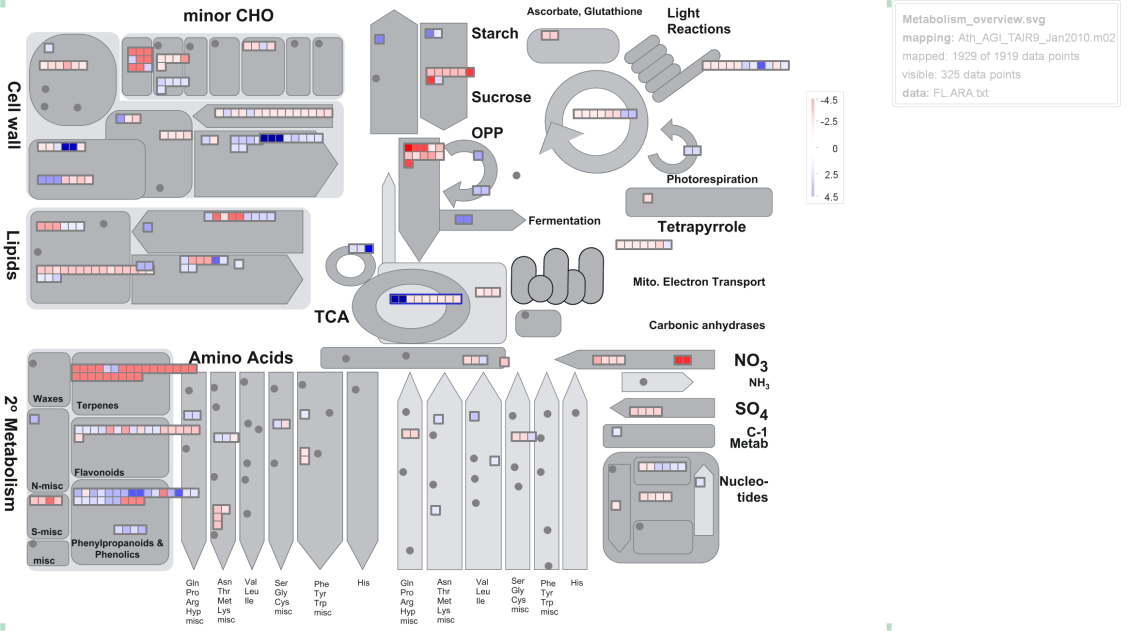

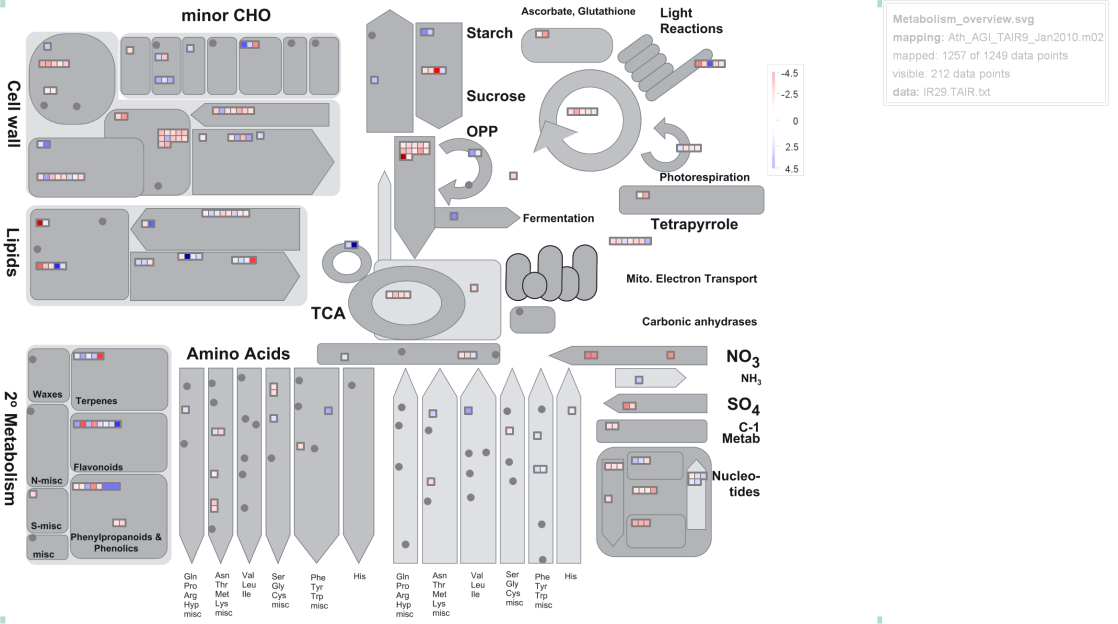


**(a)**

**(b)**

Fig S6. A visualized overview of the metabolic pathways in rice cultivars a) FL478 and b) IR29 under salt stress drown by MapMan. Color coding; red: up-regulated transcripts and blue: down-regulated transcripts.


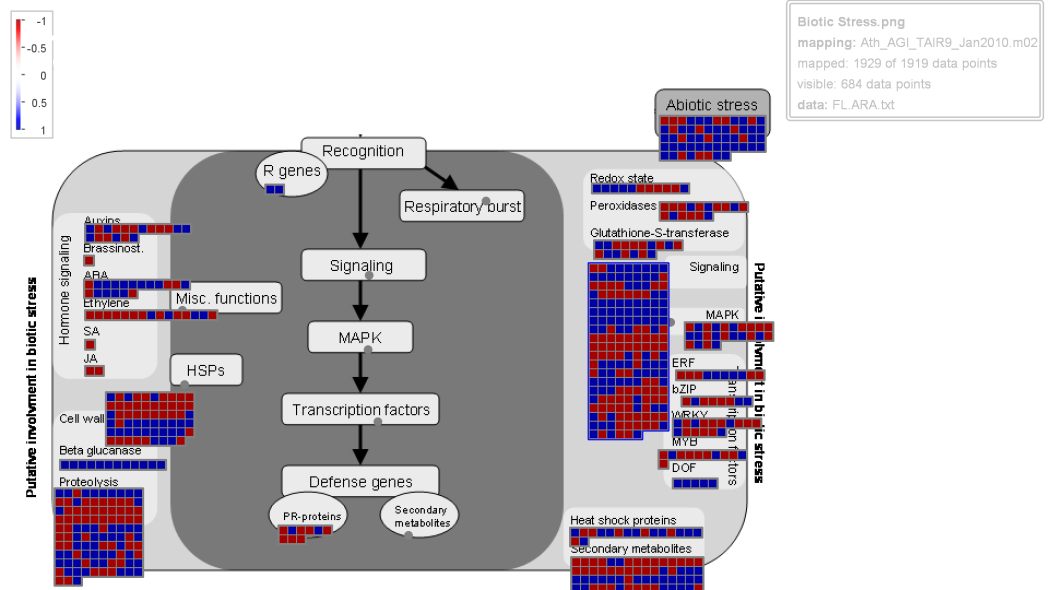

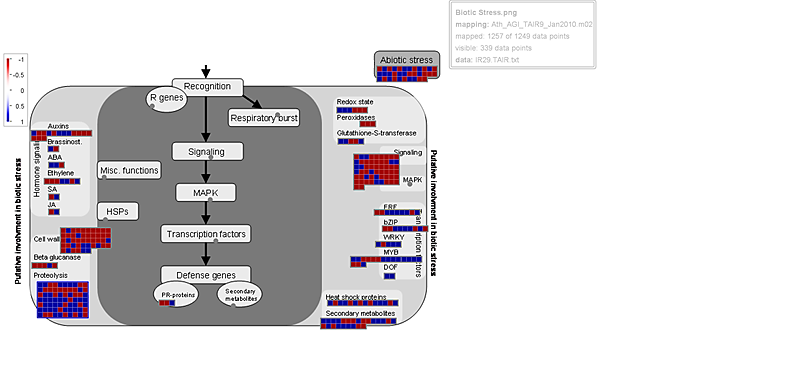


**(a)**

**(b)**

Fig S7. A graphical visualization of metabolic pathways involved in differentially expressed transcripts in a) FL478 and b) IR29 under salt stress drown by MapMan. Color coding; red: up-regulated transcripts and blue: down-regulated transcripts.


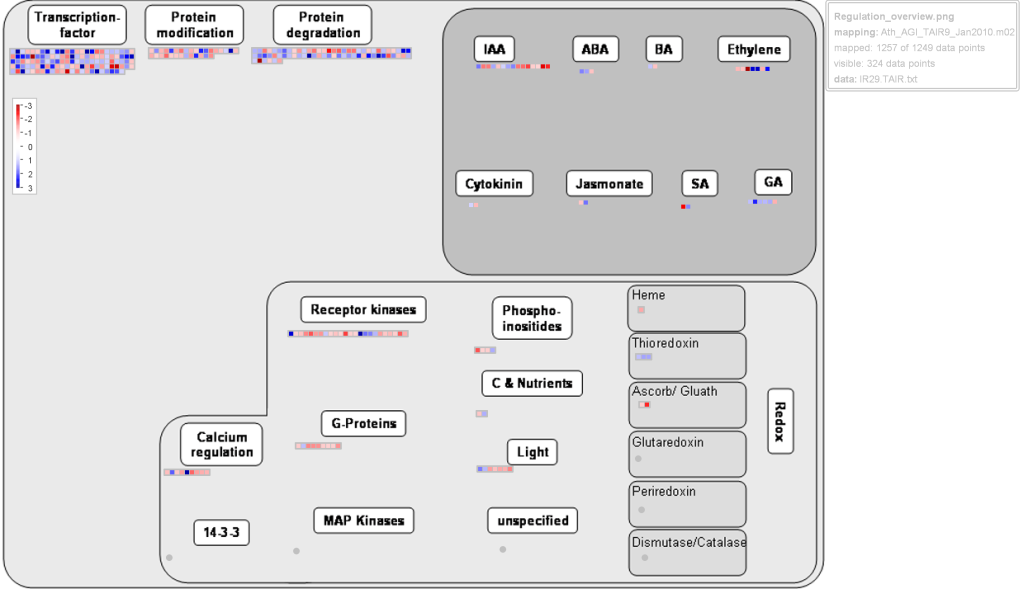

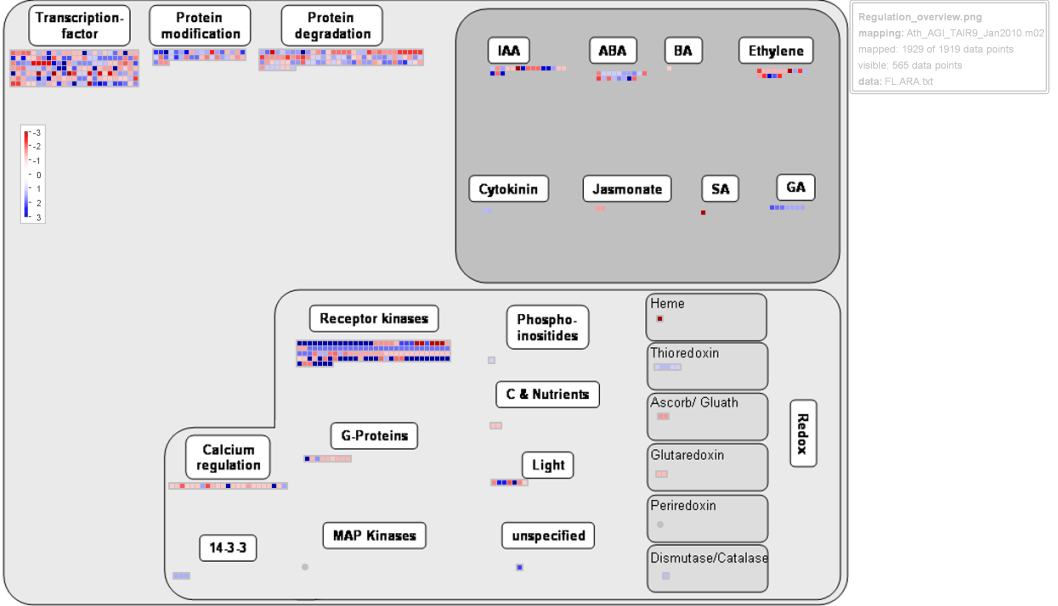


**(a)**

**(b)**

Fig S8. Schematic overview of gene regulation of differentially expressed transcripts in rice cultivars under salinity stresses, as drawn by MapMan. Various pathways were enriched in FL478 rice cultivar under salinity stress (a) and IR29 rice cultivar under salinity stress (b). Color coding; red: up-regulated transcripts and blue: down-regulated transcripts.


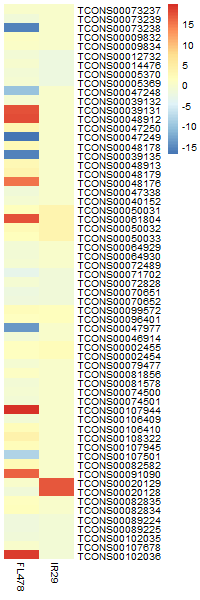

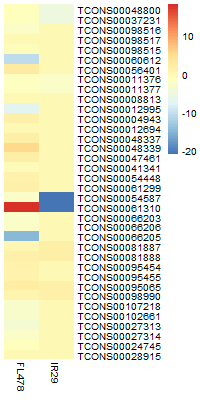

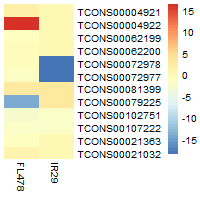

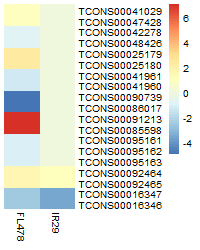


**FL478 IR29**

**FL478 IR29**

**FL478 IR29**

**FL478 IR29**

(a)Nucleic acid binding

(b)Kinase

(c) Transporter

(d) Phosphatase


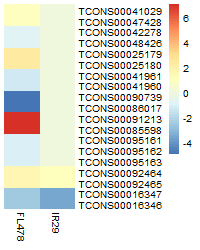


Fig S9. Differential expression of transcripts produced through intron retention (IR) event in FL478 and IR29.

**(b)**

**(a)**


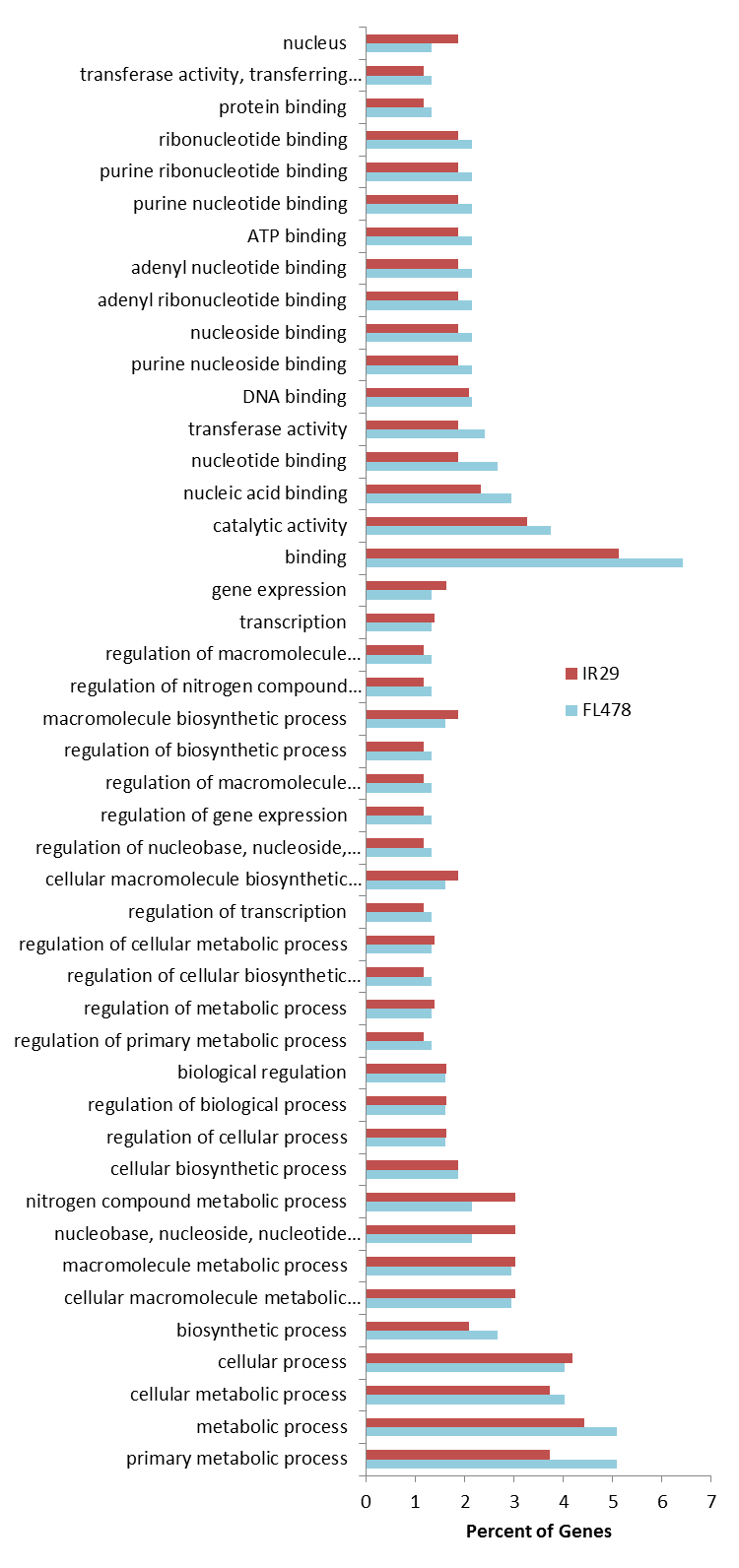

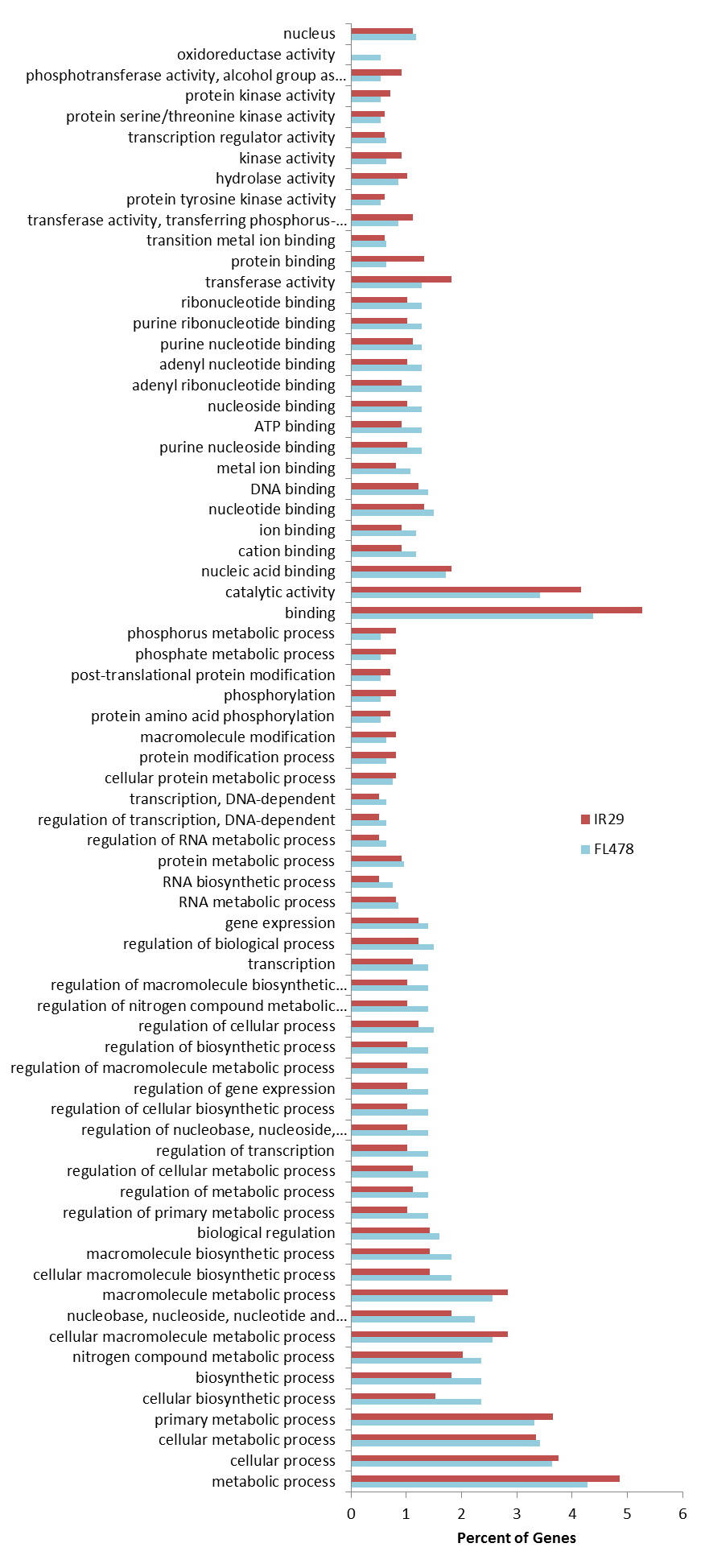


Fig S10. Geneontology enrichment statistics of transcripts produced through a) Exon skipping and b) Alternative donor in FL478 and IR29.

Table S1. Summary of sequencing results.

| IR29 | | | | FL478 | | | |  |
| --- | --- | --- | --- | --- | --- | --- | --- | --- |
| **Salinity** | | **Control** | | **Salinity** | | **Control** | | Reads mapping |
|  | |  | |  | |  | |  |
| R2 | R1 | R2 | R1 | R2 | R1 | R2 | R1 |  |
| 97.40 | 97.21 | 97.31 | 97.13 | 97.31 | 97.25 | 97.13 | 97.31 | Q20 % |
| 834778 | 952259 | 919604 | 939156 | 872343 | 1135422 | 1220890 | 1127267 | Multiple Position mapped |
| 19137593 | 13018756 | 14822137 | 14636677 | 11349364 | 12584269 | 14712544 | 14025661 | Unmapped |

Table S2. A summary of the assembly statistics.

| Parameter | FL478 | IR29 |
| --- | --- | --- |
| Total transcripts | 108633 | 109669 |
| Average transcript length (bp) | 8021 | 8243.5 |
| N50 transcript length | 2159 | 2164 |
| Transcripts with length >1000 bp | 18333 | 18638 |

Table S4. Highly enriched gene ontology (GO) terms for the differentially expressed intron retention (IR).

| FDR values in IR29 | FDR values in FL478 | Go terms |
| --- | --- | --- |
| 1.10E-152 | 4.10E-98 | P:Metabolic process |
| 2.80E-33 | 6.70E-64 | P:Cellular metabolic process |
| 3.60E-33 | 3.80E-33 | P:Biological regulation |
| 2.80E-29 | 6.20E-30 | P:Gene expression |
| 5.20E-08 | 1.40E-07 | P:Response to stress |
| 3.80E-07 | 7.70E-05 | P:Response to stimulus |
| 1.00E-22 | 6.60E-28 | P:Regulation of transcription |
| - | 4.40E-06 | P: metal ion transport |
| - | 6.00E-05 | P:cation transport |
| 2.20E-188 | 2.10E-106 | F:binding |
| 1.30E-141 | 1.10E-84 | F: catalytic activity |
| 1.70E-65 | 1.10E-42 | F: cation binding |
| 6.30E-16 | 1.20E-17 | C:nucleus |

Table S5. List of primers used for qRT-PCR analysis.

| S. no. | Gene identifier | Primer sequence |
| --- | --- | --- |
| 1 | LOC_Os01g39770.1 | F- CATCACGACAGTTTTCCCTAG |
|  |  | R-AAAGTTGGCAGGATCAGCAGG |
| 2 | LOC_Os07g08150.1 | F-ACTTGCGTATGTACTGGGTAG |
|  |  | R-CCAAGGTAGTACACCAAGCAC |
| 3 | LOC_Os03g10210.1 | F-GACTGATTAATTATGCCATGATG |
|  |  | R-AGAAGACGCCAAAACATGTGC |
| 4 | LOC_Os03g19427.1 | F-GTTGTTTGGGACGTACAGTTG |
|  |  | R-TGGCATGTTCCTCGTTTACAC |
| 5 | LOC_Os09g36200.1 | F-GGCAAGCAAATGGAAACGATC |
|  |  | R-GACGTAGACTGTAGAATGTAC |
| 6 | LOC_Os07g35004.1 | F-CTTCAACGTCAGGATCTCCAG |
|  |  | R-ACATGATTATAAACGTGCCTGC |
| 7 | LOC_Os08g38990.1 | F-TGCCGAGAGGTGAGATGAAG |
|  |  | R-ACACCATTTGTCTCATGTTCC |
| 8 | LOC_Os06g12410.1 | F-CAACAACTCCACCTCAACCTAG |
|  |  | R-GAACCAAAACAAAAACGCTGAG |
| 9 | LOC_Os02g20360.1 | F-CCAGAGGGATCAATGTTTGTG |
|  |  | R-TGATGCGAACCCAATTCTTCA |
| 10 | LOC_Os06g41010.1 | F-TTGCTGGTCACGCTGAAGTAG |
|  |  | R-GACCAACCCTCTTCCGACAAG |
| 11 | LOC_Os05g32110.3 | F-GGCAACCCAAGCTCTAATGAC |
|  |  | R-CAAGAGCATGTTGGGCAGTTC |
| 12 | LOC_Os01g62760.1 | F-GCGCAATATTCTATGCTCCATG |
|  |  | R-TATCTTGGTAGGAGCTAGTAGT |
| 13 | UBQ (internal control) | F-GCAGCATTGGACTTCATTATAC |
|  |  | R-GCACAGTGAAGCAAATTGTAC |
